# Empyema caused by *Eikenella halliae* diagnosed by metagenomic next-generation sequencing (mNGS) after pulmonary surgery: A case report

**DOI:** 10.3389/fpubh.2022.897602

**Published:** 2022-09-26

**Authors:** Jie Huang, Liming Wang, Yumei Xu, Xinhua Hu, Ronghuan Yu, Shi Chen, Baoqing Wang

**Affiliations:** ^1^Department of Respiratory Medicine, Shanghai Xuhui Central Hospital, Zhongshan-Xuhui Hospital, Fudan University, Shanghai, China; ^2^Department of Microbiology Laboratory, Shanghai Xuhui Central Hospital, Zhongshan-Xuhui Hospital, Fudan University, Shanghai, China; ^3^Department of Critical Medicine, Shanghai Xuhui Central Hospital, Zhongshan-Xuhui Hospital, Fudan University, Shanghai, China; ^4^Department of Pulmonary and Critical Care Medicine, Affiliated Hospital of Jianghan University, Wuhan, China; ^5^Department Respiratory Medicine, Zhongshan Hospital, Fudan University, Shanghai, China

**Keywords:** metagenomic next-generation sequencing (mNGS), whole genome sequencing (WGS), empyema, *Eikenella halliae*, case report

## Abstract

**Background:**

Empyema is one of the complications of pulmonary surgery for lung cancer, the incidence of which is not very high, but in severe cases, it can even lead to death, and it is always difficult to diagnose the cause by conventional methods.

**Case presentation:**

In this study, we report a clinical case of empyema caused by *Eikenella halliae* after pulmonary surgery in a 55-year-old man. He had a fever, cough, and expectoration for 3 days and was diagnosed with right hydropneumothorax and empyema, pneumonia, postoperative malignant tumor of the right lower lobe (adenocarcinoma), and hypertension. The microbiology laboratory reported Gram-negative bacteria in pleural effusion, which was preliminarily considered as *Eikenell*a based on culture and 16S rRNA sequencing. Furthermore, metagenomic next-generation sequencing (mNGS) of sputum samples was performed two times and reported negative results and the presence of *E. halliae*, respectively. The pathogen was finally confirmed as *E. halliae* by whole genome sequencing, suggesting the high-resolution ability of mNGS in the clinical diagnosis of this case.

**Conclusion:**

To our knowledge, this is the first case report of *E. halliae* infection in China, indicating increased pathogenicity of *Eikenella* sp. in immunocompromised patients, especially after invasive operations. Our findings emphasize that mNGS allows bacterial diagnosis of empyema and can significantly improve the accuracy of the diagnosis.

## Introduction

Lung cancer is one of the malignant tumors with high morbidity and mortality worldwide, especially in China (1), which in turn creates a substantial burden on society and families. Pulmonary surgery is not only the treatment for early lung cancer but also an important means to identify pulmonary shadows. Empyema refers to the presence of pus in the pleural cavity (2). As one of the complications of pulmonary surgery for lung cancer with not very high incidence, empyema may even lead to death in severe cases (3, 4). The mortality rate of empyema is 10–20% (5–7). *Staphylococcus*, particularly methicillin-resistant *Staphylococcus aureus*, and *Streptococcus* are the most important pathogens of empyema (8, 9). The current pathogen identification for empyema usually relies on direct Gram stain and routine microbiological culture (10). However, the diagnosis by conventional methods is always difficult, with 40% of cases failing to identify the etiology by culture using pleural fluid (11, 12). The poor positive rate of culture may be due to the previous antimicrobial treatment before sample collection, the low load of pathogens in the sample, and the harsh growth requirements of some pathogens.

Metagenomic next-generation sequencing (mNGS) is an advanced genomics-based technology that can theoretically detect all the nucleic acids of the specimen in one run (13, 14). It is culture-independent and can identify almost all pathogens, including bacteria, viruses, fungi, and parasites (15). In a rapid, broad-spectrum, and unbiased manner, mNGS performs significant advantages for clinical diagnosis of various diseases, especially for rare and novel pathogen infections (16). Previous studies showed that multiple samples can be used for mNGS detection, including blood and sputum (15, 17).

In this study, we reported a clinical case of empyema caused by *Eikenella halliae* after pulmonary surgery in a 55-year-old man. To our knowledge, this is the first case report of *E. halliae*-caused empyema in China, which has never been reported before. Conventional methods, mNGS, and high-through whole genome sequencing were performed to detect the pathogen.

## Case report

### Medical history

A 55-year-old male patient was admitted to Shanghai Xuhui Central Hospital due to fever, cough, and expectoration for 3 days on 9 April 2021. The patient had a history of hypertension, long-term smoking, chronic bronchitis, and emphysema. On 17 March 2021, the patient visited a tertiary general hospital in Shanghai and received a radical resection of right lower lung cancer and right upper lobectomy, and the postoperative pathology changes were various. Invasive adenocarcinoma was identified in the right lower lung, with alveolar and adherent growth, not involving the visceral pleura. A large number of acute and chronic inflammatory cells were infiltrated the right upper lobe, and fungal hyphae were specifically visible, considering fungal infections (*Candida* infection was suspected, and mucormycosis and aspergillosis were ruled out). On 7 April 2021, the patient developed a high fever, with chest computed tomography (CT) in that hospital revealing possible empyema on the right side, postoperative changes in the right lung with scattered infection, multiple fractures of the right ribs with chest wall pneumatosis, and enlarged lymph nodes in the mediastinum. His vital signs upon admission to our hospital were as follows: temperature, 39.0°C; blood pressure, 134/70 mmHg; respiratory rate, 20 breaths/min; pulse, 112 beats/min; blood oxygen saturation level (SpO_2_), 92% (without oxygen inhalation); clear mind, stable breathing, no cyanosis of lips, the disappearance of breath sounds in the right lower lung, dullness to percussion in the right lower lung, and no dry or moist rales heard. As given above, the patient was diagnosed with right hydropneumothorax and empyema, pneumonia, postoperative malignant tumor of the right lower lobe (adenocarcinoma), and hypertension.

### Hospital course

**Figure 1:**
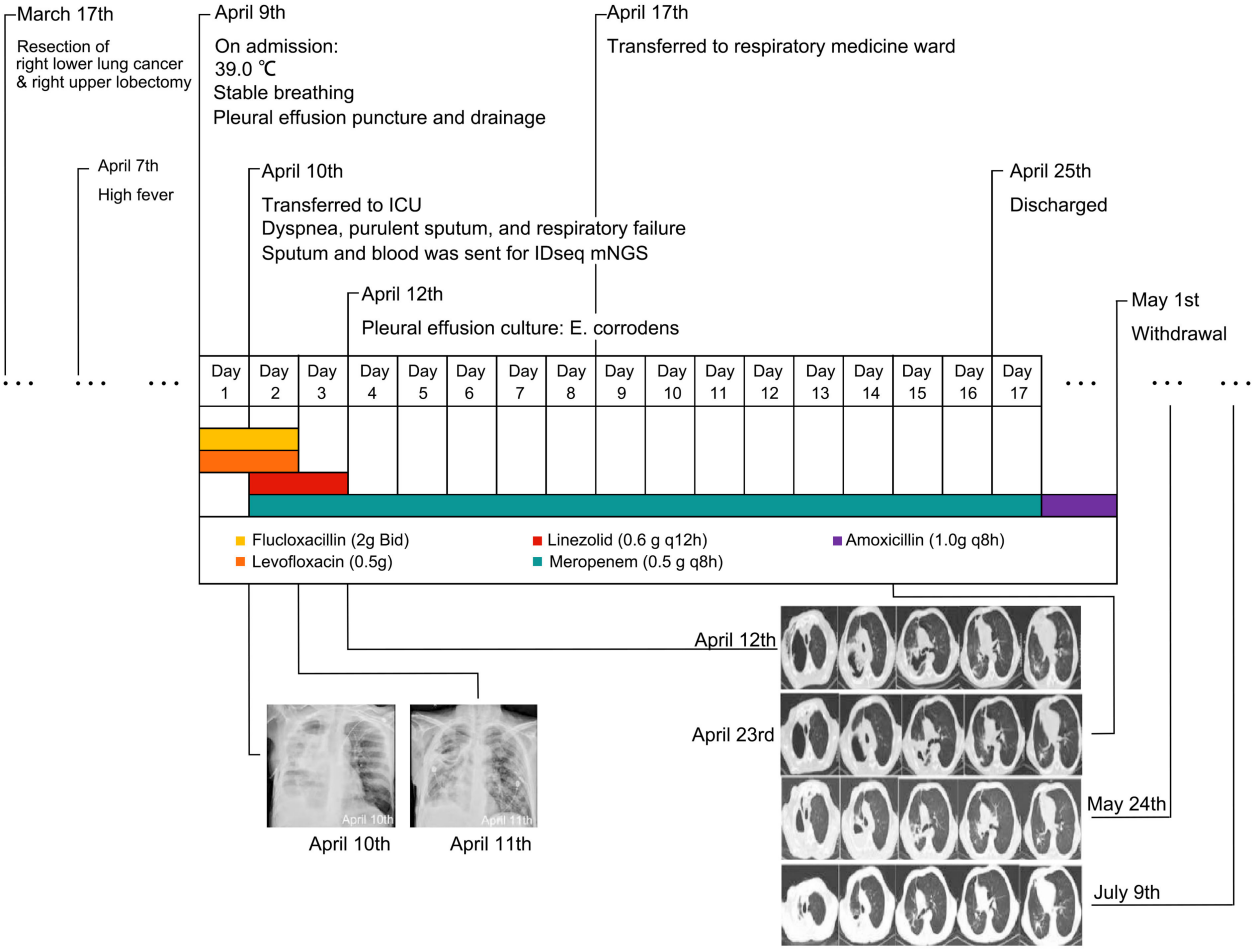
The timeline of this patient. Bedside chest x-rays on 10 and 11 April revealed patchy high-density shadows with localized consolidation in the right lung field. Chest computed tomography (CT) showed the infection was continuously absorbed.

After admission, the patient was given empirical anti-infective treatment with flucloxacillin (2 g Bid) plus levofloxacin (0.5 g Qd) injection. In addition, treatment for reducing phlegm, pleural effusion puncture and drainage, and other symptomatic treatments were given. On 10 April 2021, the patient had a sudden high fever without explained causes, accompanied by dyspnea, profuse sweating, and a large amount of purulent sputum. Physical examination revealed a heart rate of 108 bpm/min, an SpO_2_ of 75% (without oxygen inhalation), a blood pressure of 118/88 mmHg, a respiratory rate of 34 breaths/min, wheezing appearance, clear consciousness, coarse breath sounds, and a large number of palpable moist rales. Blood gas analysis showed a decreased pH of 7.237 and an elevated P_CO2_ and P_O2_ level (62.1 and 97.2 mmHg, respectively), indicating type II respiratory failure. The patient was given Bipap ventilator-assisted ventilation (A/C mode, inspired positive airway pressure (IPAP) 18 cm H_2_O, expired positive airway pressure (EPAP) 4 cm H_2_O, f = 24, O_2_ = 5 L/min) and transferred to the intensive care unit. Meropenem injection 0.5 g q8h and linezolid injection 0.6 g q12h were given immediately. The bedside chest x-ray showed infectious lesions and pleural effusion in the right lung (Figure 1, Supplementary Figure S1). The patient refused bronchoscopy. Sputum and blood samples were sent for mNGS (Vision medicals, Guangzhou, China), but both showed negative results. On 11 April 2021, the bedside chest x-ray was repeated, and the symptoms improved (Figure 1, Supplementary Figure S1). On 12 April 2021, the culture of pleural effusion in the microbiology laboratory reported Gram-negative bacteria, which was preliminarily considered as *Eikenella corrodens* based on morphology. Chest CT was performed on the same day, indicating a right lung abscess with cavitation (Figure 1, Supplementary Figure S1). The patient was given a meropenem injection of 0.5 g q8h alone for anti-infection. On 17 April 2021, the symptoms of cough, expectoration, chest distress, and shortness of breath were improved, without fever. The patient was transferred to the respiratory medicine ward. On 23 April 2021, the re-examination of chest CT showed that the lesion was continuously absorbed (Figure 1).

### Outcome and follow-up

The patient was discharged on 25 April 2021. Amoxicillin 1.0 g q8h continued for 1 week. The conditions of the patient were stable. On 24 May 2021 and 9 July 2021 (Figure 1, Supplementary Figure S1), the re-examination of chest CT showed further absorption of the lesion. To date, the patient had no fever, dyspnea, or other symptoms.

### Microbiological examination and identification

**Figure 2:**
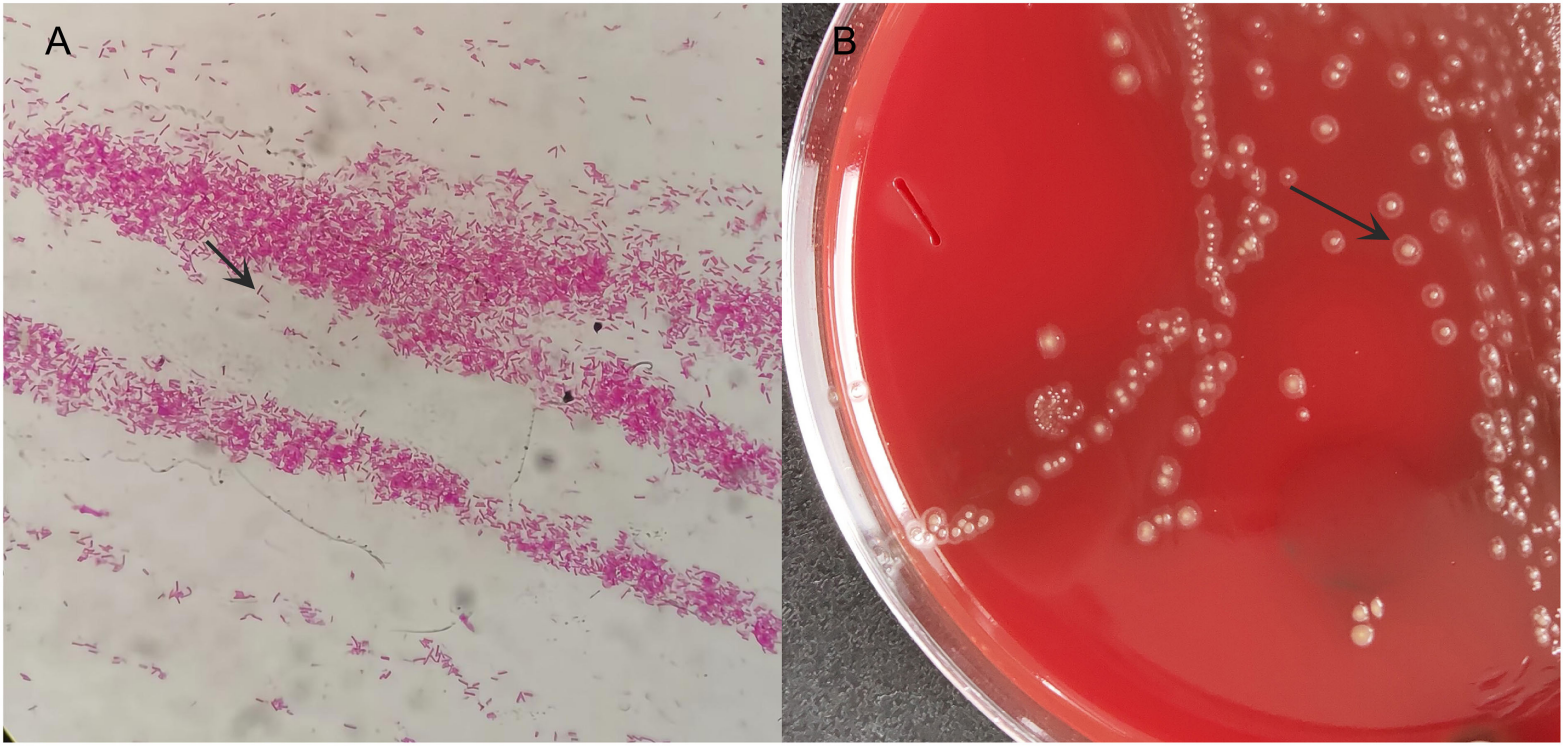
The microbiology laboratory reported positive Gram-negative bacteria and preliminarily considered *Eikenella* at the genus level. **(A)** smear Gram staining; **(B)** bacterial colony morphology on blood plate after 72 h.

Under aseptic operation, B ultrasound-guided thoracentesis was performed on 9 April 2021. A total of 15 ml of dark red pleural effusion was withdrawn and sent to the microbiology laboratory for aerobic and anaerobic culture. Direct smear staining showed elongated Gram-negative bacteria (Figure 2A). The culture was transferred to a Columbia blood agar plate and incubated at 35°C under 5% CO_2_ for 72 h. We observed a typical straw cap colony on the plate, which was flat, with central convex and smooth round (Figure 2B). A 16S rRNA gene sequencing was performed. The pathogen was detected as *Eikenella* at the genus level.

**Figure 3:**
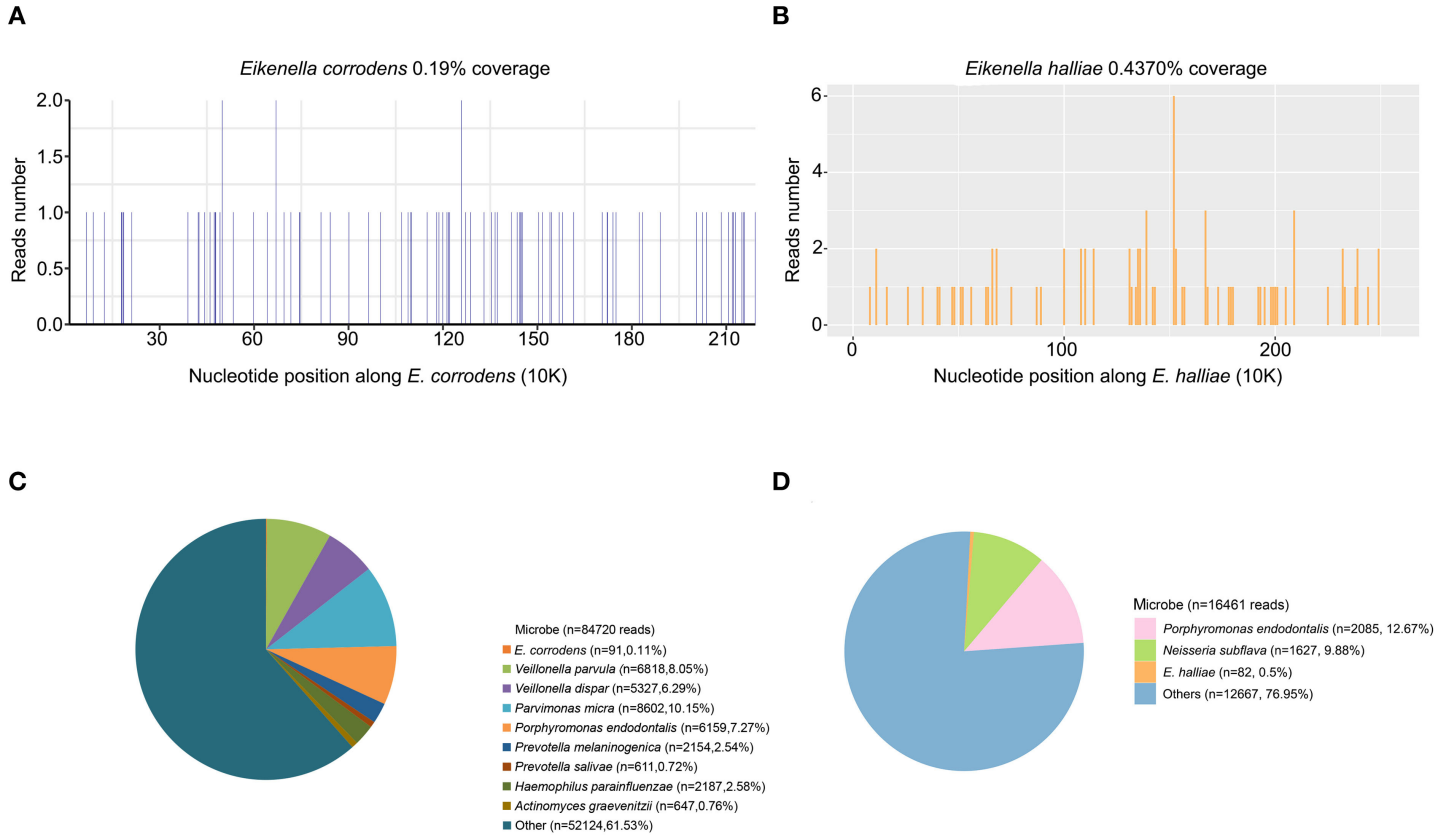
The metagenomic next-generation sequencing (mNGS) results of this patient. The coverages of *Eikenella corrodens* by the first mNGS and *Eikenella halliae* by the second mNGS are shown in **(A,B)** respectively. The detected specific read numbers and the percentages of *E. corrodens* and *E. halliae* are shown in **(C,D)**, respectively.

**Figure 4:**
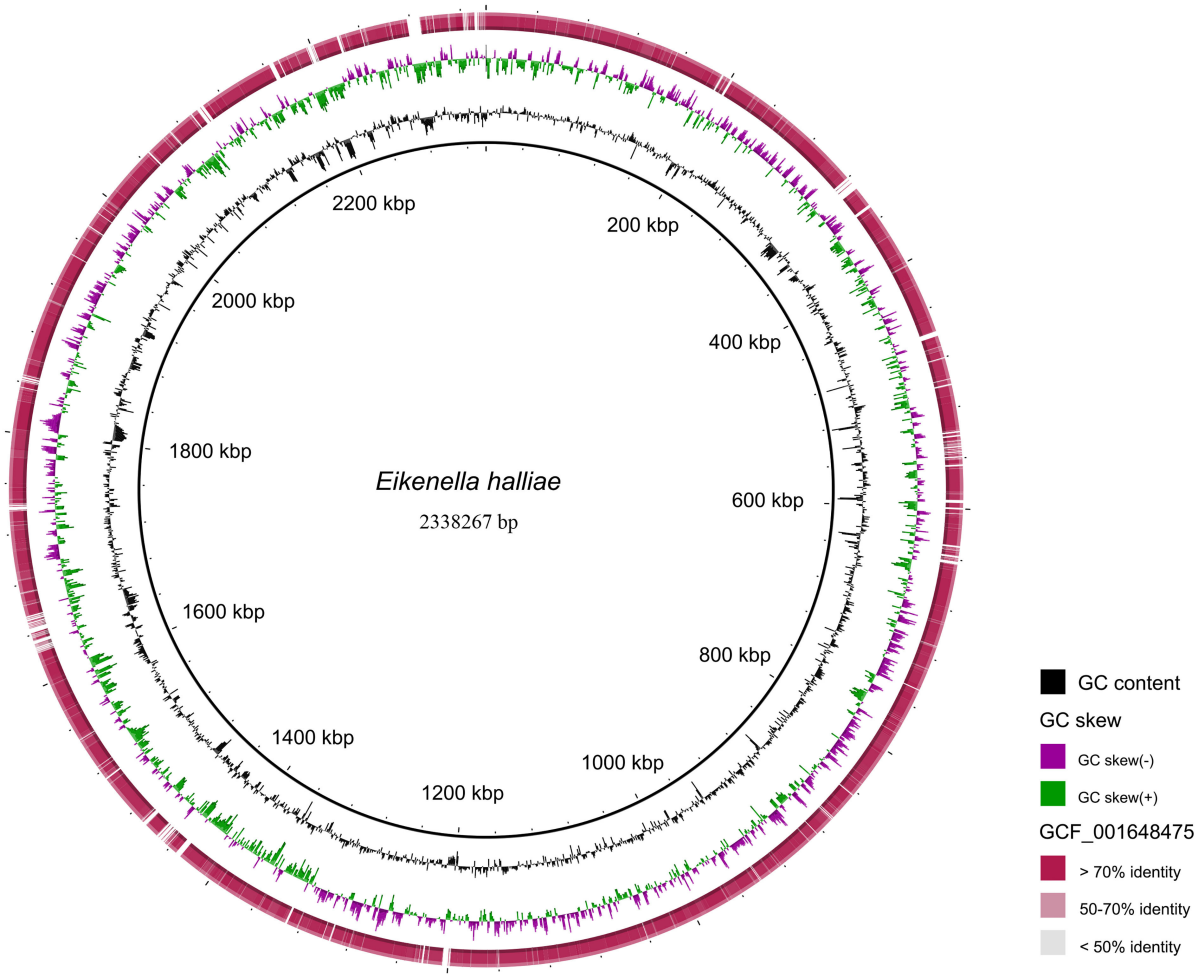
The detected NGS results of the isolate mapped to the reference genome of *Eikenella halliae* (GCF_001648475.1). Reads with >70% identity to the reference genome of *E. halliae* are marked in dark red; Reads with 50 ~ 70% identity to the reference genome of *E. halliae* are marked in light red; Reads with < 50% identity to the reference genome of *E. halliae* are marked in gray.

To further confirm the pathogen at the species level, a sputum sample collected on 11 April 2021 was sent for mNGS (Hugobiotech, Beijing, China), identifying the pathogens as *E. halliae* (82 specific reads, Figures 3B,D). Considering the rarity of the pathogen in this patient, the previously negative mNGS data were reanalyzed, and *E. corrodens* (91 unique reads, Figures 3A,C) were found in the background microorganisms. Interestingly, the pathogens detected by the two mNGS tests were different at the species level. So, high-throughput whole genome sequencing (WGS) was finally applied, of which the result showed that the isolate had the best concordance with *E. halliae* (Figure 4).

### Metagenomic next-generation sequencing (MNGS) detection

DNA was extracted from sputum samples using the QIAamp DNA Micro Kit (QIAGEN, Hilden, Germany) according to its manual. DNA libraries were then constructed by QIAseq^TM^ Ultralow Input Library Kit (Illumina, California, USA), and the quality of libraries was estimated using Qubit (Thermo Fisher, Massachusetts, USA) and Agilent 2100 Bioanalyzer (Agilent Technologies, Santa Clara, USA). The qualified libraries were finally sequenced on the Nextseq 550 platform (Illumina, California, USA). Reads of short length, low quality, and low complexity were removed from the raw data. Human DNA was also removed after mapping the human reference genome database (hg38). The remaining reads were finally aligned to the National Center for Biotechnology Information (NCBI) Microbial Genome Databases. The detailed method is shown in the Supplementary material.

### Intervention

The patient was initially treated with ß-amides and quinolones, but his conditions progressed, and respiratory failure occurred during treatment. According to the susceptibility protocol of rare bacteria and aerobic bacteria in the Clinical and Laboratory Standards Institute (CLSI) M45-A3 (18) document, this bacterium was highly sensitive to amoxicillin/clavulanic acid. To further determine the sensitive drugs, the broth microdilution method was used for the susceptibility test in this case, and it was found that amoxicillin/clavulanic acid was the best antibiotic drug for the patient.

## Discussion

*Eikenella* mostly colonizes on the mucosal surface, but it can also invade the surrounding tissues and cause infection. Previous studies revealed that it can cause oral infection, respiratory tract infection, liver abscess, neck abscess, meningitis, conjunctivitis, osteomyelitis, etc., but the incidence is low worldwide (19).

To the best of our knowledge, this was the first case of *E. halliae* infection in China. The patient's condition was complicated. He previously received radical resection of right lower lung cancer and right upper lobectomy at the same time. One possible cause for postoperative empyema after pulmonary surgery is the decreased immune function of the patient. Additionally, both the patient's history of smoking and chronic airway diseases, such as chronic bronchitis, emphysema, daily cough, and expectoration, and postoperative incision pain affecting normal respiratory movement and limiting cough and expectoration, could cause poor drainage of airway secretions, further complicated with pulmonary infection. Second, pulmonary infection foci could invade the pleural cavity *via* the lymphatic spread. Besides, pulmonary surgery is a contaminated surgery with postoperative chest drains routinely left in place, and the pleural cavity and pleural effusion may become the culture medium, which could also cause postoperative empyema (20). Many cases with chest infections due to *Eikenella* had a history of lung cancer (21, 22). However, the occurrence of chest infections caused by *E. halliae* after lung surgery was reported for the first time. This mentioned the increased pathogenicity of *Eikenella sp*. in an immunocompromised patient with cancer, especially after invasive operations.

Culture is often considered to be an important standard for the diagnosis of infectious diseases, which can not only provide an etiological basis but also provide the basis for the selection of antibacterial drugs. In this case, due to the deterioration of the patient's condition after the initial empirical treatment, it is necessary to consider rare bacterial infections. *E. halliae* is a Gram-negative and compatible anaerobic bacterium with high nutritional requirements. It takes 48–72 h for the typical colony morphology to form (23), similar to the culture results in this case. The identification of *E. halliae* by culture is difficult; in this case, the microbiology laboratory could only identify it as *Eikenella*. The limitations are obvious.

The metagenomic next-generation sequencing (mNGS) technique emerges as an alternative and efficient molecular diagnostic method. However, there are some challenges. In this case report, *E. corrodens* was detected but considered as background microorganisms by the first mNGS detection, while the second mNGS diagnosed the pathogen as *E. halliae*, which was then confirmed by the WGS of the isolates. The difference between the two mNGS results may be due to the different reference databases and bioinformatics analysis methods. This indicated that optimized reference databases and bioinformatics analysis methods of mNGS should be further explored, and a standard for mNGS detection is needed.

After the pathogen is identified, sensitive antibacterial drugs are selected for treatment according to the susceptibility results, the patient's condition is improved, and the lesions are significantly absorbed during follow-up. The successful experience of diagnosis and treatment in this patient also shows that it is important to fully master the characteristics of clinical infection of *E. halliae*, carry out strain identification, and select sensitive drugs for timely and accurate clinical diagnosis and treatment to avoid misdiagnosis and ineffective treatment.

We report the first case, to the best of our knowledge, of empyema due to *E. halliae*, which was diagnosed by mNGS. This indicated that *E. halliae* could be a potential pathogen in immunocompromised patients with cancer after invasive operations. mNGS performed a great advantage in diagnosing rare and fastidious pathogens.

## Data availability statement

The datasets presented in this study can be found in online repositories. The names of the repository/repositories and accession number(s) can be found below: http://ngdc.cncb.ac.cn, PRJCA007766.

## Ethics statement

This study was approved by the Ethics Committees of Shanghai Xuhui Central Hospital. The patients/participants provided their written informed consent to participate in this study. Written informed consent was obtained from the individual(s) for the publication of any potentially identifiable images or data included in this article.

## Author contributions

JH and BW developed the study design. JH, LW, YX, XH, RY, and SC collected clinical information. BW provided supervision throughout this project. JH wrote the manuscript. All authors interpreted the data and read, reviewed, and approved the manuscript.

## Funding

This work was supported by the Key Medical Specialty of Shanghai (ZK2019C14), the Medical Research Project of Xuhui District, Shanghai (SHXH202006), and the Special Clinical Research Project of Shanghai Municipal Health Commission (202140473).

## Conflict of interest

The authors declare that the research was conducted in the absence of any commercial or financial relationships that could be construed as a potential conflict of interest.

## Publisher's note

All claims expressed in this article are solely those of the authors and do not necessarily represent those of their affiliated organizations, or those of the publisher, the editors and the reviewers. Any product that may be evaluated in this article, or claim that may be made by its manufacturer, is not guaranteed or endorsed by the publisher.
