# Supplementary figures and images for: Empyema caused by Eikenella halliae diagnosed by metagenomic next-generation sequencing (mNGS) after pulmonary surgery: A case report
Source: Front Public Health. 2022 Sep 26;10:897602. doi: 10.3389/fpubh.2022.897602 (PMC9550235; doi:10.3389/fpubh.2022.897602)

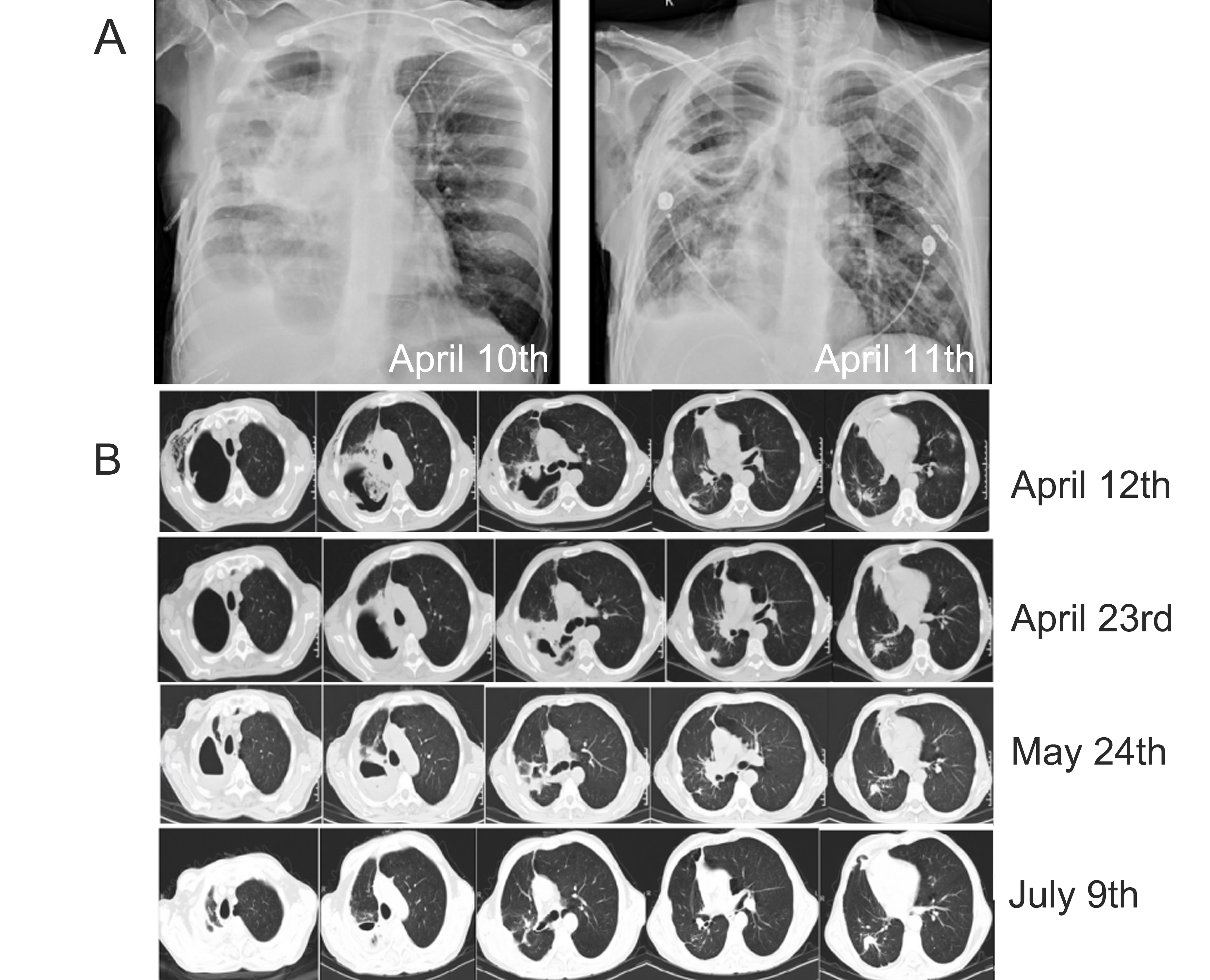

Supplement: Supplementary file 1 [file Image_1.jpg]
